# Assessing ethical practices among community pharmacists in Nigeria: prevalence, drivers, challenges, and implications—a mixed methods study

**DOI:** 10.3389/fpubh.2025.1495328

**Published:** 2025-05-13

**Authors:** Olanrewaju Olamide Popoola, Igbagboyemi Adebowale Adebisi, Adebowale Sylvester Adeyemi, Damilola Samson Adepitan, Opeyemi Fortunate Oladeru

**Affiliations:** ^1^HealthPlus, Lagos, Nigeria; ^2^Faculty of Pharmacy, Obafemi Awolowo University, Ile-Ife, Nigeria; ^3^Drugfield Pharmaceuticals, Ogun, Nigeria; ^4^Arkland Health Limited, Abuja, Nigeria; ^5^University of Ilorin, Ilorin, Nigeria; ^6^University College Hospital, Ibadan, Nigeria

**Keywords:** pharmacy practice, community pharmacists, antimicrobial resistance, substandard and falsified medicines, drivers, ethics, challenges

## Abstract

**Introduction:**

Given the accessibility of community pharmacists as the first point of care in Nigeria and their expanding role in optimizing public health, it is critical that they employ evidence-based, ethical practices. This study assessed ethical practices and identified drivers of unethical practices among community pharmacists in Nigeria.

**Methods:**

A convergent-parallel mixed method study design was employed, collecting quantitative and qualitative data, which were analyzed using SPSS and inductive content analysis.

**Results:**

Most pharmacists maintained patients’ confidentiality (93.2%), and disclosed side effects of medicines to patients (80.3%). Nearly half (48.9%) dispensed antibiotics without prescriptions and more than one-third purchased medicines from the unregulated open market (38.4%). Conversely, only 53.4% performed quality checks to identify possible or suspected substandard and falsified medicines. Pharmacists who regularly consulted the PCN code of ethics were less likely to dispense antibiotics without prescriptions (*p* = 0.011), stock medicines not approved by NAFDAC (*p* = 0.010), or purchase from open markets (*p* = 0.027). Key drivers of unethical pharmacy practices include poor physician-pharmacist interprofessional collaboration (76.3%), patient pressure (73.9%), and managerial pressure to meet sales targets (70.3%). Qualitative responses highlighted competitive pressure, expressed as fear of losing clients, poor working conditions, poor regulation, and unethical, profit-driven, managerial practices as drivers of unethical pharmacy practices. Potential limitations to this study include recall bias and the Hawthorne effect.

**Conclusion:**

Pharmacists’ unethical practices potentially contribute to the prevalence of substandard and falsified medicines, and antimicrobial resistance. Improved regulations, improved remuneration, providing incentives for professionalism, training on ethics and improved multidisciplinary collaborations could promote ethical community pharmacy practices.

## Introduction

The pharmacy profession has continuously evolved from merely dispensing to providing comprehensive pharmaceutical care, optimizing medicine use, and improving patient health outcomes (1). Community pharmacies are a vital expression through which pharmacists deliver pharmaceutical care. Community pharmacists play a crucial role in optimizing medicine use, conducting medicine reviews, providing health and medicine information to patients and healthcare professionals, administering vaccinations, offering point-of-care testing, processing prescriptions, ensuring optimal dosage regimens and formulations, identifying potential drug–drug or drug-food interactions, dispensing and disposing of medications, detecting substandard and falsified medicines, preventing and managing non-communicable diseases (NCDs), ensuring adequate medicine supply and appropriate medicines storage (1, 2). Through these activities, pharmacists promote patients’ optimal health and well-being, prevent adverse effects, and ensure medication effectiveness (1).

Pharmacists are the most accessible and trusted healthcare professionals (3), and the ease of accessing them, coupled with the robust services they provide, has established them as the primary point of contact for medical services in Nigeria (4). Nigeria’s population of 220 million is being serviced by an estimated 13,000 practicing pharmacists (5). This estimate amounts to approximately one pharmacist per 17,000 Nigerians, a great shortfall from the World Health Organization’s (WHO) recommendation of at least 3 pharmacists per 10,000 population (6). In Nigeria, due to inadequacies in primary healthcare provision, long waiting times in hospitals, and shortages of medical practitioners, community pharmacies often function as primary healthcare centers, providing comprehensive, coordinated, and continuous primary care to patients (4). As the role of community pharmacists in providing healthcare services in Nigeria continues to grow, it is critical to empower them to practice ethically. A community pharmacy operates as both a business (required to generate economic value and profit to thrive) and as an expression of pharmacy practice that must uphold ethical and professional standards. Maintaining the balance between pharmacy ethics and profitability can be challenging, leading to conflicting decisions and ethical dilemmas that may compromise the standard of community pharmacy practice in Nigeria (7, 8).

Ethical pharmacy practice refers to pharmacy practice that aligns with the highest level of professional conduct that prioritizes the optimal interest of the patient and community at large (9). Unethical pharmacy practice like the sale of antibiotics without prescription contributes to antimicrobial resistance (AMR), may cause side effects, reduce the effectiveness of medicines, compromise patient safety, and contribute to poor health outcomes (1, 10). Additionally, by purchasing medicines that are not quality-certified or purchasing medicines from unregulated markets, community pharmacists can contribute to the prevalence of substandard and falsified medicines, which could result in adverse effects, increased healthcare costs, and mortality (11).

Ethical practice in Nigeria is regulated by the Pharmacy Council of Nigeria (PCN), a federal government parastatal vested with the mandate to oversee and govern all facets and ramifications of pharmaceutical education, training, and professional practice, as well as, the regulation for pharmacy technicians and patent and proprietary medicine vendors (PPMVs) (12). PPMVs are defined by the PCN as “*a person without formal training in pharmacy who sells orthodox pharmaceutical products on a retail basis for profit”* (12). While PPMVs are permitted to only sell over-the-counter medicines, they are not authorized to dispense prescription-only medicines due to their lack of formal training in pharmacy and medicines (12). Poor regulation has contributed to the unethical dispensing of antibiotics without prescriptions in both community pharmacies and PPMVs (10), contributing to antimicrobial resistance. Furthermore, the National Agency for Food and Drug Administration and Control (NAFDAC) is responsible for regulating the quality, distribution, sale, manufacture, use, and disposal of medicines in Nigeria (13). Several limitations and challenges like poor funding, inadequate staff, judicial bureaucracies, and corruption encountered by NAFDAC in executing its duties contribute significantly to the proliferation of substandard and falsified medicines in Nigeria undermining good pharmacy practice (14, 15).

Several researchers have investigated the ethical challenges and dilemmas among pharmacists in community pharmacies in many countries of the world. Benson and colleagues (16) observed that ethical challenges among community pharmacists in the United Kingdom were caused by conflicting obligations between personal values, ethical guidelines, and managerial rules. This study identified that the lack of simulated practical session on ethics training may have contributed to the community pharmacists not handling ethical dilemmas correctly. A study in India reported similar findings (17), stating that conflicting objectives of upholding ethics, fulfilling clients request and management’s wishes contributed to unethical practices in India. Participants in this study also reported that maintaining ethical standards in rural settings was challenging, particularly because many patients lacked education on pharmacy ethics. They noted that practicing ethically could sometimes be perceived by patients as limiting their autonomy in making healthcare decisions. Additionally, they found it easier to uphold ethical standards when dealing with patients who already trusted them than newer patients. Pharmacists also faced ethical dilemmas in maintaining confidentiality when discussing cases with colleagues as some believed that sharing patient information with other professionals could improve health outcomes Furthermore, they highlighted conflicts between professional regulatory compliance and addressing patient needs in emergency situations. The fear of losing customer loyalty was another significant concern, as pharmacists worried that strict adherence to ethical guidelines might negatively impact patients’ loyalty.

Chaar (18) reported that inadequate training on professional ethics, limited ethical practice among community pharmacists in Australia. Community pharmacists in this study emphasized that maintaining ethics served the customer’s best interests. However, a key challenge was balancing professional obligations with commercial pressures. They often faced dilemmas where business priorities, like profitability and sales targets, conflicted with their ethical duty to prioritize patient care. Participants further highlighted that maintaining patient confidentiality in an era of digital health records and increasing data-sharing requirements was challenging. Workplace constraints, like high workload and time pressures, further hindered pharmacists from engaging in thorough patient counseling and ethical decision-making. These challenges, as highlighted in literature, underscore the complexities of ethical decision-making in pharmacy practice. Moreover, the expanding role of community pharmacists in patient care makes it even more critical for them to uphold ethical standards and professionalism.

Nevertheless, there is a dearth of literature focused on ethics in pharmacy practice in Nigeria. Some researchers assessed non-prescription sales of antibiotics in Northern Nigeria but did not investigate other unethical practices (19). Okoro (20) identified poor knowledge of ethical principles among hospital and community pharmacists in Nigeria, however, they did not examine their ethical practices or identify ethical dilemmas. There is a paucity of scholarly inquiry evaluating the holistic ethical pharmacy practice and identifying factors that precipitate unethical practices among community pharmacists in Nigeria. Therefore, this research’s overarching objective is to address this gap. This research aims to assess ethical pharmacy practice, identify salient drivers that contribute to unethical conduct, and propose potential solutions to reinforce adherence to professional ethics among community pharmacists in Nigeria.

## Methods

### Study design

A cross-sectional survey was conducted from January to March 2024 using a self-administered questionnaire. The survey instrument included both quantitative and qualitative questions. Utilizing a convergent-parallel mixed method study design, this study collected both quantitative and qualitative data simultaneously. This study design offered the advantage of comparing, analysing, and integrating data from both the quantitative and qualitative responses. Additionally, this study design enabled us to investigate the subjective, in-depth experiences of community pharmacists in Nigeria and identify economic and social factors that influence unethical pharmacy practice.

### Sample population

Our study included community pharmacists practicing across Nigeria. Only community pharmacists who were fully registered with the Pharmacy Council of Nigeria and have practiced in community pharmacy for at least 6 months were included in the research.

### Sample size determination

The minimum sample size was calculated using the Raosoft software with a confidence level of 90% and an error margin of 5%, with 5,380 community pharmacists practicing in Nigeria (5), yielding 258 responses.

### Sampling

Convenience sampling was used to select participants (21).

### Data collection tool and validation

The questionnaire was developed strictly using the ethical requirements as stated in the Pharmacy Council of Nigeria’s (PCN) Code of Ethics to assess community pharmacy practice in line with the national standard (12). The PCN code of ethics provides ethical guidelines on pharmacists’ conduct when interacting with patients, other professionals, the community, the supply chain, and performing professional conduct.

The 26-item questionnaire covered five domains gathering data on demographics (9 questions) including pharmacy qualification, age, years of experience; ethical pharmacy practice during professional communications with patients and other health professionals (6); ethical pharmacy practice while providing pharmaceutical care (4); ethical pharmacy practice while interacting with the supply chain (5); common reasons for unethical pharmacy practice as reported in literature (8, 22, 23). and an open-ended question on reasons for unethical practices. Responses to practice questions were collected across a 5-point Likert scale for frequency from “always” to “never.” While responses on common reasons for unethical practice were collected on a 5-point Likert scale from “strongly agree” to “strongly disagree.”

The questionnaire was piloted with twenty experienced community pharmacists. This pilot provided feedback that contributed to reframing the initial survey questions, to avoid leading questions, and validated the survey instrument. Additionally, it ensured that the survey instrument was suitable for collecting data that answered our research questions. During the pilot, we assessed the reliability of the questionnaire by using the Cronbach alpha test of reliability. The Cronbach’s alpha was 0.75 on a 53-item scale, indicating the questionnaire has an acceptable reliability score.

### Data collection

Data was collected using a self-administered, validated, web-based, semi-structured questionnaire using Google forms. The survey instrument was distributed through the Pharmaceutical Society of Nigeria Young Pharmacists Group (PSN-YPG). The Pharmaceutical Society of Nigeria (PSN) is a non-regulatory association of pharmacists in Nigeria that oversees pharmacists’ welfare (24). Concurrently, the Association of Community Pharmacists of Nigeria (ACPN) was also involved in the survey distribution and data connection.

### Data analysis

Descriptive data analyses were conducted using IBM SPSS (V.25) to report frequencies of ethical/unethical practices. The Pearson Chi-square test was used to determine if years of experience, highest pharmacy qualification (B. Pharm, M. Pharm, or PharmD), pharmacy management or frequency of reference to the pharmacy code of ethics statistically influenced ethical pharmacy practice (*p*-value > 0.05). Qualitative responses from the open-ended questions were analysed using inductive content analysis. The analysis process commenced with open coding, wherein the data was meticulously examined to get familiar with the responses. Afterward, codes were assigned to capture the relevant categories in the responses of the participants. These codes (subthemes) were inductively derived, emerging organically from participants’ responses, reflecting the participants’ perspectives and experiences. The coding process was iterative, with constant comparison between subthemes to ensure they were mutually exclusive, exhaustive (capturing all relevant data), consistent, and accurate. Subsequently, subthemes that referred to or addressed the same issue were classified into broader categories called themes. All themes and codes identified emerged organically from the data collected from responses. This method offered the advantage of a systematic and transparent analytical process that ensures the reporting of valid and reliable inferences based on participants’ responses alone. Participants were anonymised and given numbers. Additionally, words in square brackets were introduced to give context to participants’ responses.

### Theoretical paradigm

This research employed an interpretive phenomenological paradigm to evaluate individual subjective experiences in community pharmacy practice. This subjective perspective enabled us to understand pharmacists’ lived experiences with ethical dilemmas in community pharmacy practice. Furthermore, it enabled us to examine ethical pharmacy practice through the viewpoint (lens) of the participants and develop themes that gave a true understanding of the drivers of unethical pharmacy practices and propose practicable solutions to mitigate these practices in Nigeria.

### Positionality

Some researchers, OFO and DSA, which conducted this research were currently practicing as community pharmacists in Nigeria at the time of this research. POO, AS, and AI have previously worked as community pharmacists in Nigeria. To mitigate potential biases arising from previous experiences, reflexivity was employed throughout the research process. This involved consciously examining, appraising, and assessing how our prior experiences might influence the analysis and interpretation of responses. Furthermore, the phenomenological paradigm employed reduced the risk of bias as it enabled the researchers to separate their own experience from that of the respondents. Also, the codes and themes generated were reviewed by two independent researchers to validate the consistency of the codes generated. Mitigating bias through reflexivity, and external validation of coding strategies enhanced the credibility and reliability of this research (25).

### Ethical consideration

The study was conducted in compliance with the PCN’s code of conduct and ethical standards. Participants understood the research, voluntarily agreed to participate, and could withdraw at any time during the data collection process. All responses were anonymised and informed consent was given by all participants before participating in the research. All activities conducted in this research comply with the Declaration of Helsinki as reviewed in 2013.

## Results

Two hundred and nineteen (219) responses were received from 25 states for this survey, bringing the response rate to 85%. Most respondents 193 (88.1%) had a Bachelor of Pharmacy degree, 80 (36.5%) respondents had 1–3 years of experience, and 79 (36.1%) respondents were locum pharmacists (see Table 1). On the other hand, 48 (22%) worked in pharmacies owned by non-pharmacists.

**Table 1:** Demographic information.

Item	Frequency	Percentage (%)
Age		
<25 years	35	16.0
25–34 years	158	72.1
35–44 years	21	9.6
>45 years	5	2.3
Total	219	100.0
Highest qualification		
B. Pharm	193	88.1
M. Pharm/Masters	13	5.9
PharmD	13	5.9
Total	219	100.0
Years of experience		
6 months-1 year	47	21.5
1–3 years	80	36.5
3–5 years	48	21.9
>5 years	44	20.1
Total	219	100.0
Is the pharmacy you work in owned by a pharmacist?		
Yes	171	78
No	48	22
State of practice		
Abia	1	0.5
Akwa-Ibom	7	3.2
Anambra	17	7.8
Bauchi	1	0.5
Bayelsa	1	0.5
Benue	1	0.5
Borno	1	0.5
Cross-River	6	2.7
Delta	14	6.4
Edo	12	5.5
Ekiti	1	0.5
FCT Abuja	35	16.0
Imo	3	1.4
Kaduna	1	0.5
Kano	3	1.4
Kogi	1	0.5
Kwara	3	1.4
Lagos	58	26.5
Nassarawa	3	1.4
Niger	3	1.4
Ogun	5	2.3
Ondo	6	2.7
Osun	14	6.4
Oyo	12	5.5
Plateau	2	0.9
Rivers	7	3.2
Yobe	1	0.5
Total	219	100.0

**Figure 1:**
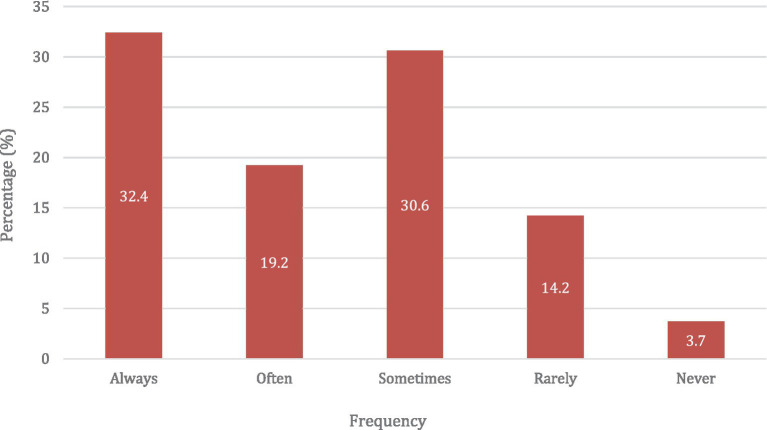
Frequency of reference to the PCN code of ethics in decision-making.

The survey responses revealed that 183 (83.6%) use the Ethical Code of Ethics as the official document to guide professional ethics recommended by the PCN. However, only 51.6% always or often referred to it to guide their decisions (see Figure 1). The majority of the respondents, 193 (88.1%) reported having a private consultancy room in the pharmacy.

Regarding ethical practice during patient communications, most respondents reported a strong commitment to professional ethics. When responding to the statement “I offer honest professional advice and counseling to patients even when they may not like it,” 90.9% (64.4% always and 26.5% often) of respondents reported consistently doing so. Similarly, patient confidentiality was highly prioritized, with 93.2% (77.2% always and 16% often) of respondents reporting they consistently ensured confidentiality during private consultations and other duties. When responding to the statement “I disclose possible side effects of medicines to patients when dispensing,” 80.3% (56.6% always and 23.7% often) of respondents reported consistently disclosing side effects of medicines to clients on dispensing. Regarding the prompt referral of patients with advanced medical cases to a hospital, 91.3% (68.5% always and 22.8% often) of respondents reported adhering to this practice. In response to “I readily collaborate with other health professionals to ensure optimal health outcomes,” 88.6% (63.5% always and 25.1% often) of respondents expressed a strong interest in multidisciplinary collaboration. Conversely, when asked about monitoring and reporting adverse drug reactions through pharmacovigilance forms, only 43.8% (26% always and 17.8% often) reported consistently engaging in pharmacovigilance activities (see full breakdown of responses in Table 2).

**Table 2:** Responses to questions assessing ethical practice.

Questions	Responses [frequency (%)]				
	Always	Often	Sometimes	Rarely	Never
Questions to assess ethical pharmacy practice during professional communication with patients and health professionals.					
I offer honest professional advice and counseling to patients even when they may not like it	141 (64.4)	58 (26.5)	19 (8.7)	1 (0.5)	0 (0)
I ensure patients’ confidentiality and private consultation in executing my duties	169 (77.2)	35 (16)	13 (5.9)	2 (0.9)	0 (0)
I disclose possible side effects of medicines to patients when dispensing.	124 (56.6)	52 (23.7)	26 (11.9)	9 (4.1)	8 (3.7)
I promptly refer health cases beyond my knowledge and expertise to the hospital.	150 (68.5)	50 (22.8)	14 (6.4)	4 (1.8)	1 (0.5)
I readily collaborate with other health professionals to ensure optimal health outcomes.	139 (63.5)	55 (25.1)	17 (7.8%)	7 (3.2)	1 (0.5)
I monitor and report adverse drug reactions through the pharmacovigilance form.	57 (26)	39 (17.8)	51 (23.3)	41 (18.7)	31 (14.2)
Questions to assess ethical pharmacy practice while providing pharmaceutical care					
I make pharmaceutical care decisions based on laboratory result	56 (25.6)	92 (42)	63 (28.8)	6 (2.7)	2 (0.9)
I dispense antibiotics to patients without a prescription when they demand it.	7 (3.2)	18 (8.2)	30 (13.7)	52 (23.7)	112 (51.1)
I dispense medicines likely to be abused by clients when they demand it.	3 (1.4)	3 (1.4)	6 (2.7)	27 (12.3)	180 (82.2)
I dispense medicines that I suspect may be falsified and/or substandard.	4 (1.8)	2 (0.9)	5 (2.3)	15 (6.8)	193 (88.1)
Questions to assess ethical pharmacy practice while interacting with the supply chain					
I purchase, store, or/and dispense medicines not accredited by NAFDAC	4 (1.8)	5 (2.3)	48 (21.9)	55 (25.1)	107 (48)
I purchase medicines from the open market	3 (1.4)	6 (2.7)	40 (18.3)	35 (16)	135 (61.6)
I dispose of expired and damaged medicines through NAFDAC	77 (35.2)	35 (16)	36 (16.4)	29 (13.2)	42 (19.2)
I maintain the cold chain during the purchase, transportation, and storage of temperature-sensitive medicines	148 (67.6)	39 (17.8)	18 (8.2)	9 (4.1)	5 (2.3)
I perform quality control checks on medicines and pharmaceutical devices purchased to identify substandard and/or falsified medicines.	80 (36.5)	37 (16.9)	39 (17.8)	38 (17.4)	25 (11.4)

Turning to pharmacy practice while providing pharmaceutical care, in response to “I make pharmaceutical care decisions based on laboratory results,” 67.6% (25.6% always and 42% often) of respondents reported consistently prioritizing laboratory-based decisions. When responding to “I dispense antibiotics to patients without a prescription when they demand it,” 51.1% of respondents reported never and 23.7% rarely dispensing antibiotics without a prescription when they demand them. Additionally, in responding to “I dispense medicines likely to be abused to clients when they demand it,” the majority, 82.2% of respondents, reported never and 12.3% rarely engage in such acts. Similarly, almost all respondents, 94.9% (88.1% never and 6.8% rarely) refrain from dispensing medicines suspected to be substandard or falsified.

Examining pharmacy practice while interacting with the supply chain, in response to the statement “I purchase, store, or/and dispense medicines not accredited by NAFDAC,” majority 73.1% (48% never and 25.1% rarely) of respondents reportedly refrained by patronizing non-NAFDAC accredited medicines. Similarly, in responding to the statement on purchasing medicines from the open market, 61.6% of respondents reported never and 16% rarely purchasing medicines from the open market. When asked about medicines disposal practices through NAFDAC, only 51.2% (35.2% always and 16% often) reported to disposing expired or damaged medicines through NAFDAC. Regarding the statement “I maintain the cold-chain during the transportation and storage of temperature-sensitive medicines,” 85.4% (67.6% always and 17.8% often) of respondents reported ensuring cold chain is maintained during the purchase and transportation of temperature-sensitive medicines. Conversely, only 53.4% (36.5% always and 16.9% often) reported performing quality checks on medicines and pharmaceutical devices to identify substandard or falsified medicines (see Table 2 for responses to all statements).

Respondents agreed or strongly agreed that poor physician-pharmacist inter-professional collaboration (76.3%), patients pressure (73.9%), managerial pressure to meet sales targets (70.3%), poor communication channels to reach doctors (69.9%), bad patient behavior (66.7%), financial incentives to meet sale targets (65.7%), poor regulation from the PCN (61.2%), poor remuneration (56.6%), poor knowledge of drug disposal channels (55.3%), and poor incentive for professionalisms (53.9%) are among the main drivers of unethical pharmacy practice in Nigeria.

The data in Table 3 presents the report of the Pearson Chi-Square test showing how factors like frequency of reference to the code of ethics, highest qualification, years of experience and pharmacy ownership influenced pharmacy practices. Findings revealed a statistically significant association (*p* < 0.05) between how frequently pharmacists refer to the PCN Code of Ethics and their ethical pharmacy practices in Nigeria.

**Table 3:** Analysis of ethical pharmacy practice based on relevant factors.

Relevant factors	Question (ideal ethical response)			
	I dispense antibiotics to patients without a prescription when they demand It (Never).	I purchase, store, or dispense medicines not accredited by NAFDAC (Never).	I purchase medicines from the open market (Never)	I monitor and report adverse drug reactions through the pharmacovigilance form (Always).
Frequency of referring to the PCN code of ethics
Always	67.6%	69.0%	70.4%	50.7%
Often	52.4%	50.0%	57.1%	23.8%
Sometimes	40.3%	37.3%	58.1%	14.9%
Rarely	38.7%	32.3%	61.2%	0.0%
Never	37.5%	25.0%	25.0%	12.5%
*p-*value	0.011	0.010	0.027	<0.001
Highest qualification				
B. Pharm	53.4%	49.2%	60.1%	27.5%
M. Pharm/Masters	38.5%	53.8%	69.2%	7.7%
PharmD	30.8%	38.5%	76.9%	23.1%
*p*-value	0.236	0.909	0.732	0.232
Years of experience				
6 months-1 year	59.6%	57.4%	61.7%	31.9%
1–3 years	51.2%	43.8%	60.0%	22.5%
3–5 years	50%	47.9%	70.8%	22.9%
>5 years	43.2%	50%	54.5%	29.5%
*p*-value	0.281	0.193	0.608	0.436
Pharmacy ownership				
Pharmacist owned	52%	48%	64.9%	25.7%
Non-pharmacists owned	47.9%	52.1%	50%	27.1%
*p*-value	0.644	0.655	0.051	0.797

### Qualitative responses

Regarding the qualitative analysis, 101 pharmacists responded to the open-ended questions providing saturated data for evidence-based conclusions. Responses on the factors that limited ethics pharmacy practice were classified into 17 subthemes which finally formed 4 larger themes (See Table 4 for description of themes and subthemes).

**Table 4:** Analysis of qualitative responses.

Theme	Subtheme	Description	Additional quotes
Non-professional management of pharmacy.		The unprofessional management of community pharmacies creates an environment where ethical standards of the pharmacists are compromised for business interests	
	Unethical managerial directives	This occurs when Pharmacy owners and managers explicitly instruct pharmacists to engage in unethical practices	*“Poor managerial instructions on the part of the pharmacy owner” R75.*
	Pressure from management to make sales	This occurs when management imposes sales targets that compel pharmacists to compromise professional standards in order to meet business requirements, get bonuses or avoid negative consequences.	*“There is pressure from the boss to boost sales” R206.* *“The idea of meeting* [sales] *target should be scraped out to promote ethics” R87.*
	Lack of managerial awareness of professional ethics	This occurs when non-pharmacist owners or managers who lack an understanding of ethical guidelines create environments where unethical practices flourish.	*“Non-pharmacists who do not know about ethics are handling the business and setting up Pharmacies” R56.*
	Intrusion in pharmacists’ decision making	This occurs when pharmacists face interference from non-pharmacist staff, owners, managers, or family members who override or undermine their professional judgment, limiting their autonomy to make ethical decisions	*“Pharmacy owners should stop family interference in running of Pharmacy” R54.* *“For pharmacies owned by a pharmacist, the manager who might not be a pharmacist tends to have more influence in the decision-making” R162.*
	Poor inventory management	This occurs when poor inventory management, including purchasing near-expiry medications, insufficient stocking of essential medicines, and punishing pharmacists for expired products, puts pharmacists under pressure to compromise ethical standards	*“They* [the management] *are deducting from my salary due to expired drugs” R37.*
Social and economic (Socioeconomic) pressure		Refers to external pressures (outside the community pharmacy) from the broader socioeconomic environment that may force community pharmacists to choose between maintaining ethical standards and responding to market demands, patient demands, and competitive pressures	
	Patient pressure	This occurs when community pharmacists face pressure from patients to engage in unethical practices	*“So, the fact that you are doing the right thing and not letting the patient have their way even after proper explanation, the patient would tell you if you do not sell to them, they can always get it elsewhere” R18.*
	Poor economic conditions	This occurs when the challenging national economic environment and lack of financial support for pharmaceutical operations limits community pharmacists’ ability to maintain profit while adhering to ethical standards	*“The government needs to prioritize and invest in healthcare” R83.*
	Competitive pressure	This occurs when community pharmacists face intense competition from other pharmacies and PPMVs, who may not adhere to ethical practices.	*“Patent medicine stores operate without a code of ethics, hence when you professionally counsel a patient on why POM should not be dispensed without prescriptions, they simply go to patent stores to purchase them…” R49.*
Poor working conditions		Suboptimal working conditions that compromise pharmacists’ ability to maintain ethical practices.	
	Inadequate staffing	This occurs when understaffing forces pharmacists to undertake multiple roles simultaneously, compromising their ability to provide optimal pharmaceutical care.	*“Work overload. In many community pharmacies, the pharmacist does his/her work in conjunction with being a cashier, manager” R147.* *“Better modalities should be put in place to ensure that professionals are on the ground in every pharmacy premises. All premises should allow sufficient space for private consultations” R41.*
	Hostile management-staff relationship	This occurs when negative workplace interactions between management and pharmacists cause strain, psychological stress, or a toxic work environment that compromises pharmacy ethics.	*“Lack of a conducive work environment for pharmacists compromises ethical practice” R121.*
	Poor remuneration	This occurs when pharmacists are not fairly paid, and receive unsatisfactory or untimely salaries, demotivating them to uphold ethical standards.	*“The employers should do better in terms of salary for community pharmacists” R124.* *“Lack of prompt payment of salary is another issue” R94.*
	Lack of incentive for professionalism	This refers to the absence of recognition, rewards, or motivation to maintain high ethical standards.	*“There is a lack of incentive for good professional conduct” R55.*
	Lack of basic infrastructure	This occurs when the lack of essential infrastructure in the pharmacy (including constant electricity, storage facilities, diagnostic kits, inventory management software, or other facilities) limit ethical practice.	
Poor regulation		Inadequate enforcement of regulations and ethical guidelines that allows unethical practices to persist.	
	Lack of Retribution for Unethical Practices	The absence of consistent enforcement of penalties for violation of ethical standards.	*“Perhaps more punishment is needed for defaulters” R22.*
	Pharmacies operating without pharmacists	This occurs when pharmacies operate without qualified pharmacists present, enabling unqualified personnel to take up pharmacists’ roles in patient care.	*“Poor pharmacy inspection to ensure that only qualified professionals [pharmacists] are in charge of dispensing medicines and counseling” R39.*
	Poor regulation of PPMVs	This occurs when there is inadequate monitoring and enforcement of standards for PPMVs permitting them to dispense prescription-only medicines	*“There should be all-round regulation that does not only control pharmacists but also involve all other stakeholders* [including PPMVs]” *R57.*
	Influence of pharmaceutical companies	This occurs when pharmacists face conflicts of interest due to financial ties with pharmaceutical companies	

#### Theme 1: Non-professional management of pharmacy

Pharmacists identified that the non-professional management of community pharmacies contributed to unethical practices. The non-professional management of community pharmacies encompassed five subthemes: unethical managerial policies and directives, managerial pressure to make sales, non-pharmacist ownership of community pharmacies, intrusion in pharmacists’ decision-making, and poor-inventory management.

Subtheme 1: Unethical managerial directives.

Some community pharmacists reported being directed by their managers to engage in unethical practices.

*“Some pharmacy owners direct us [community pharmacists] to sell antibiotics to patients without prescription when they ask” R38*

Subtheme 2: Pressure from the management to make sales.

Some community pharmacists mentioned that the pressure from the management to make a profit and meet sales targets was a primary reason for compromising adherence to ethical standards. Others identified that managers were more profit-oriented than ethically conscious.

*“…major thing is the sales target from the management, it is becoming crazy in Nigeria” R72.*

*“Pharmacy managers are often more business-oriented than ethically driven” R61.*

Subtheme 3: Lack of managerial awareness of professional ethics.

Pharmacists reported that the ownership or management of pharmacies by non-pharmacists, who may be unaware of ethical guidelines, contributes to unethical practices. These managers, due to their lack of knowledge, may disregard guidance on pharmaceutical care and ethics.

*“Non-pharmacist directors or managers feel they know better when you give guidance on pharmaceutical care and ethics …” R 165.*

*“The lack of understanding on the part of the pharmacy owner that pharmacy practice has ethical rules is also a factor” R175.*

Subtheme 4: Intrusion in pharmacists’ decision making.

The lack of autonomy for pharmacists in decision-making within the pharmacy is a significant limitation. Interference from non-pharmacy support staff or managers can lead to frustration and compromised ethical practices. As respondents stated,

*“The intrusion of pharmacist assistants in some pharmacies affects proper ethical pharmacy practice” R5.*

*“The pharmacist should be in charge of managing POM [Prescription-only-medicines] and not the pharmacy owner” R38.*

Subtheme 5: Poor inventory management.

Managers who are untrained in inventory management may purchase short-dated medicines, which are often cheaper, and pressure pharmacists to sell them. Additionally, some managers fail to adequately stock the pharmacy but still expect pharmacists to sell the available medicines to maximize profits. Lastly, the deduction of salary from pharmacists due to medicines expiry was highlighted. These can lead to unethical dispensing of medications that patients may not need, as pharmacists expressed:

*“Short-dated [near expiry] products should not be bought to put pressure on the pharmacists to sell them” R87.*

*“Medicines stock out issue, when the pharmacy does not have the exact medication the client needs, but you still have to make the most of what you have on the shelf to maximise profit for the organization” R19.*

#### Theme 2: Social and Economic (Socioeconomic) pressure

Socioeconomic pressures can be divided into three subthemes: pressure from the patients, bad economic conditions, and competitive pressure not to lose market value.

Subtheme 6: Patient pressure.

Respondents noted that they sometimes succumb to patients’ pressure to maintain relationships and meet societal expectations, which can lead to unethical practices.

*“Patients overly exaggerate their confidence in you and sometimes we do it for them to manage … sometimes at their insistence” R34.*

Subtheme 7: Poor economic condition.

The challenging economic conditions in the country, coupled with a lack of financial support for pharmaceutical operations, contribute to difficulties in maintaining ethical practices.

*“The bad Economy and paucity of financial aids to assist pharmaceutical operations contributes to the problem …” R27.*

Subtheme 8: Competitive pressure.

Competitive pressure from other pharmacists and PPMVs who do not practice ethically forces some pharmacists to sell POMs without prescriptions. Pharmacists who uphold professional ethics may risk losing sales and customer loyalty as patients may bypass ethical pharmacists and purchase POMs from other stores instead.

*“Well, it is now a community challenge if you do not sell, the next pharmacy would. A Patient walks across and purchases prescription-only medicines from a patent medicine store if you refuse to sell” R22.*

*“… another thing that may contribute to compromise is if other colleagues are not doing the right thing, your client may end up going to that other pharmacy if they don’t get the antibiotic or other POMs they may be asking for” R 185.*

#### Theme 3: Poor working conditions

Ethical dilemmas could also originate from poor working conditions encompassing five subthemes: hostile management-staff relationships, poor remuneration, lack of incentive for professionalism, inadequate staffing, and lack of basic infrastructure compromising ethical pharmacy practice.

Subtheme 9: Inadequate staffing.

Inadequate staffing was highlighted as a significant challenge in many pharmacies, limiting pharmacists’ ability to provide optimal pharmaceutical care and engage ethically with patients. Due to understaffing, pharmacists often have to take on multiple roles, such as cashier, accountant, and manager, which causes stress, limits the time available for patient consultations, and increases workload.

*“Inadequate manpower, sometimes, the pharmacist is the only employee in the pharmacy and does the job of an accountant, cashier, and attendant, which limits time for patient handling …” R128.*

*“Owners of community pharmacy should provide other domestic staffs like cleaners, accountants etc. In order to reduce unnecessary stress on the pharmacist” R128.*

Subtheme 10: Hostile management-staff relationships.

A respondent highlighted that hostile management-pharmacist relationships create an environment that limits adherence to ethical standards.

*“The way people [management] talk to their staff [pharmacist] has made it impossible for them to concentrate on their work and this may cause them not to do their work the way it is supposed to be” R 202.*

Subtheme 11: Poor remuneration.

A participant noted that the *“nationwide pathetic salary structure of pharmacists” R21* contributes to unethical practices, reflecting the dissatisfaction of community pharmacists with their current wages. A respondent highlighted the need for a minimum wage or salary to be established, especially for pharmacists working in private pharmacy settings. Additionally, the lack of prompt payment of salaries further exacerbates the issue.

*“I believe there should be a minimum wage or salary for a pharmacist especially those working for private Pharmacy premises” R184.*

Subtheme 12: Lack of incentive for professionalism.

The lack of incentives, such as recognition and rewards for ethical practice, reduces motivation for pharmacists to maintain ethical standards and professionalism.

*“There needs to be incentives to be law-abiding regarding ethical practice” R57.*

Subtheme 13: Lack of basic infrastructure.

The lack of reliable electricity hampers pharmacy operations, particularly in maintaining the cold chain for temperature-sensitive medications, as rising fuel costs make it difficult to power refrigerators.

*“The non-availability of electricity to power refrigerators for maintaining the cold chain [is a challenge], especially as fuel costs have become increasingly expensive" R193.*

#### Theme 4: Poor regulation

This theme emerged from four subthemes: lack of retribution for unethical practices, pharmacies operating without pharmacists, poor regulation of PPMVs, and influence of pharmaceutical companies.

Subtheme 14: Lack of retribution for unethical practices.

The lack of enforcement of consequences for unethical practices continues to be a major concern in maintaining ethical standards. Respondents emphasized the need for stronger enforcement of regulations by regulatory agencies to ensure conformity in practice.

*“I want to urge regulatory agencies to be up and doing in enforcing laws [including sanctions] so as to ensure conformity in ethical practice” R60.*

*“There is no punishment for unethical behavior” R55.*

Subtheme 15: Pharmacies operating without pharmacists.

Here, respondents identified the need to enforce regulations ensuring that qualified pharmacists, not technicians, oversee drug dispensing and counseling.

*“PCN should ensure there is always a pharmacist in every premise attending to patients and not technicians” R196.*

Subtheme 16: Poor regulation of PPMVs.

Respondents identified that the lack of regulation of PPMVs contributes to unethical practices flourishing and called for improved monitoring of all stakeholders in the pharmaceutical system to ensure safer ethical practices across the sector.

*“The lack of regulation of PPMVs allows unethical practices and quackery to thrive, PPMVs should have stricter rules of operation and be monitored properly” R175.*

Subtheme 17: Influence of Pharmaceutical Companies.

A respondent highlighted that financial ties with pharmaceutical companies create conflicts of interest that may bias community pharmacists’ ethical decision-making.

*“Pharmacist should be unbiased in their decisions and should not be influenced by financial ties to pharmaceutical companies” R16.*

## Discussion

This study aimed to assess ethical pharmacy practices and identify factors that contribute to unethical conduct among community pharmacists in Nigeria. Overall, the findings of this study suggest that the majority (over four-fifths) of community pharmacists in Nigeria maintained strong ethical communication with patients. They provided honest professional advice to patients, ensured patients’ confidentiality, disclosed side effects of medicines to patients on dispensing medicines, and ensured proper cold chain management for temperature-sensitive medicines. However, about half of them unethically dispensed antibiotics without prescription, potentially compromising patient safety and contributing to AMR. Additionally, less than half regularly engaged in pharmacovigilance activity, reporting adverse effects, and just above half always or often performed quality checks on medicines to detect substandard and falsified medicines (Table 2).

Analysis and comparison of both the quantitative and quantitative responses revealed that unethical community pharmacy practice in Nigeria is driven by economic, profit-driven motives that undermine professional practice such as managerial pressure to meet sales targets and financial incentives tied to sales. Secondly, systemic issues such as poor regulation from the PCN, poor communication channels to doctors, limited implementation of sanctions for unprofessionalism, and poor economic conditions were also highlighted as factors that limit professionalism and ethical conduct. Thirdly, poor working conditions such as poor remuneration of pharmacists, lack of incentives for professionalism, hostile staff/management relationship, and inadequate staffing of Pharmacies contribute to unethical practices. Lastly, knowledge of appropriate drug disposal channels and pressure from the customers undermine professional ethics.

Regarding the ethical provision of pharmaceutical care, pharmacists in this study unethically dispensed antibiotics without prescriptions. The non-prescription dispensing of antibiotics in this study was 48.9%, lower than the 73.3% reported in China (26), and 88% in Mozambique (27). Perhaps the observed discrepancies could be due to the difference in the methodological approaches, with both studies exclusively utilizing qualitative study designs, simulated client method, and in-depth interviews, respectively. On the other hand, the findings of this study are similar to a mixed method study (semi-structured questionnaire followed by in-depth interviews) conducted in Ambo, Ethiopia that reported non-prescription dispensing of antibiotics to be 43.1% (28). Our findings were similar to a nationwide survey in Nigeria that reported non-prescription use of antibiotics to be 47.7% (10).

Reports from this study highlight that years of experience, pharmacy qualification, non-pharmacist ownership of pharmacy, and age were not statistically associated (*p*-value> 0.05) with non-prescription dispensing of antibiotics, as this practice was common with pharmacists in all groups. This contrasts with findings from Sudan (29), India (17), and Northern Nigeria (19), which reported that pharmacists with more than five years of experience practiced more ethically than those with less than 5 years of experience. Discrepancies with Abubakar and Tangiisuran (19) could be attributed to this study’s larger sample size and broader reach, gathering responses from over 25 states in Nigeria as opposed to just two states. The differences with findings from India (29) might be due to their longer data collection period of 9 months.

A study (10) identified that antibiotics commonly dispensed over the counter to patients for self-medication, amoxicillin (amoxil), ciprofloxacin (ciprotab) and metronidazole (flagyl), reported higher trends of antibiotic resistance in Nigeria, suggesting that the practice of non-prescription dispensing of antibiotics contributes to the prevalence of AMR in Nigeria (10).

While interacting with the supply chain, most pharmacists, over four-fifths, never or rarely purchased, stored, or dispensed medicines not accredited by NAFDAC, however, a minority, 4.1%, engage in this act. Since the quality and authenticity of non-NAFDAC-accredited medicines are not guaranteed, their use increases the risk of exposure to substandard and falsified (SF) medicines, exacerbating the public health challenge posed by SF medicines in the country (30). The abstinence of most, over 80%, pharmacists (see Table 2) from purchasing, storing or dispensing non-NAFDAC-accredited medicines highlights their commitment to maintaining the integrity of the medicine supply chain, which is crucial in safeguarding public health. It also highlights the important role community pharmacists play in upholding NAFDAC’s standards, ensuring that only medicines meeting established criteria and verified quality are dispensed to protect public health. Although the majority, more than four-fifths, of respondents reported that they would not dispense any medicine suspected to be substandard or falsified, only about half always or often perform quality checks on medicines to identify SF medicines. The practices of pharmacists in this study contradict the opinions reported by Adigwe (30), which highlighted that 98.5% of pharmacists acknowledge their responsibility to assess medicines for quality and report SF medicines. Not assessing purchased medicines for quality or authenticity limits the detection of SF medicines. Additionally, the poor engagement of less than half of the pharmacists in this study in pharmacovigilance limits further investigation into the quality of medicines dispensed to ascertain the causes of side effects. This is important as batches of substandard or falsified medicines are often identified after reported cases of adverse drug effects (31). These acts by community pharmacists potentially promote the sale and distribution of substandard and falsified medicines, contributing to treatment failure, adverse effects, increased healthcare costs, and antimicrobial resistance (11).

AMR in Nigeria is a major public health challenges as it is associated with over 263,000 deaths in Nigeria (32). Pharmacists can contribute to mitigating AMR, by ensuring optimal, evidence-based use of antimicrobials; promoting patient education on AMR to limit self-medication with antimicrobials; improve supply chain integrity by assessing quality medicines and identify substandard and falsified medicines; and involving in pharmacovigilance to report adverse drug reactions which would help identify substandard or falsified medications that contribute to AMR. To promote the evidence-based use of medicines, community pharmacists can partner with physicians through telemedicine to promote virtual consultations with doctors and the use of e-prescriptions. This cost-effective method will reduce cost and time associated with visiting the doctor and may reduce the non-prescription dispensing of antibiotics. The desire for multidisciplinary collaboration in this study was very high as over four-fifths of respondents indicated they are readily willing to collaborate with other health professionals, including doctors. Furthermore, equipping pharmacists with C-reactive protein kits can help distinguish between viral and bacterial infections, which may help reduce the inappropriate dispensing of antibiotics for viral infections like cold and flu (33). These kits are cost-effective, easy to use, and were observed to reduce non-prescription sale of antibiotics by 15% in a study in Awka, Nigeria (33). Pharmacists as the most accessible and trusted health professionals (3), play a pivotal role in informing patients of the dangers of inappropriate antimicrobial use. Patient education has been highlighted as an effective strategy to correct public misconceptions about the effectiveness of antibiotics to treat all health issues, which often drive patient demand for antibiotics without prescriptions (10, 33).

Community pharmacists can contribute to maintaining the integrity of the supply chain by refraining from purchasing non-NAFDAC accredited medicines, maintaining cold-chain for temperature-sensitive medicines, and purchasing medicines from accredited suppliers only, not patronizing the open market which is a major source of substandard and falsified medicines (14). Some of the challenges with detecting and reporting SF medicines by pharmacists in low-and middle-income settings include a lack of awareness of how to detect and report them, and lack of appropriate testing equipment (31). Community pharmacists can detect substandard and falsified medicines by examining the packages for spelling errors, checking expiry dates, verifying product composition, and conducting physical inspections of products for changes in smell, colour, or solubility (11, 31). Physical examination to detect substandard and falsified medicines is cost-effective and time-efficient, requiring limited technical equipment or laboratory setup, providing a practical approach to identifying potential threats without significant financial or time investment. Additionally, the PCN should enhance awareness of platforms for reporting substandard and falsified medicines to facilitate timely and efficient data collection, helping to address the issue more effectively.

The underreporting of adverse drug events by community pharmacists in this study may stem from several factors. Unlike hospital settings with established reporting structures, community pharmacists often lack institutional structures for pharmacovigilance. Community pharmacists also face time constraints, insufficient training on reporting procedures of pharmacovigilance, and may perceive reporting as burdensome due to the paperwork with little visible impact (34). Implementing multifaceted solutions is crucial to addressing these challenges. Pharmacovigilance reporting of adverse effects should be digitalised to reduce the time burden of paper-based reporting. Also, community pharmacists need to be educated and sensitized on the importance of pharmacovigilance and how it contributes to medication safety to motivate participation. Finally, fostering collaborative networks between community pharmacies and regulatory bodies may strengthen the entire pharmacovigilance system and improve patient safety outcomes.

Pharmacists who always referred to the PCN code of ethics to guide professionalism were less likely to sell antibiotics without prescription (*p* value = 0.011), likely to purchase or store medicines not accredited by NAFDAC (*p*-value = 0.010), less likely to purchase medicines from the open market (*p*-value = 0.027) and more likely to engage in pharmacovigilance activities (*p*-value < 0.001) than those who never refer to the code of ethics to guide professionalism (see Table 3). This evidence supports that encouraging pharmacists to regularly reference the code of ethics when in doubt of professional conduct may improve ethical practice.

The drivers of unethical practices identified in this study align with the findings of previous studies that highlighted a profit-oriented approach to community pharmacy practice, compromising patient safety (17, 22). Li and colleagues (35) also highlighted that potential gaps in pharmacists’ knowledge of ethical principles influence the prevalence of dispensing non-prescription antibiotics. Additionally, the pressure imposed by managers to meet sales targets, or prioritize profit over patient wellbeing resonates with reports by Ancuceanu (7) who discussed the tension between business needs and ethical obligations in community pharmacies. Similarly, competitive pressure, expressed as the fear of losing customers, poor remuneration and poor regulation were reported by Li and colleagues (35) as a driver of unethical dispensing practices.

This study’s findings suggest several key recommendations to improve community pharmacy practice. To begin with, the government needs to make grants, tax-relief and zero interest loans available to community pharmacists. This may potentially ease the cost of operating the pharmacy and reduce the pressure to compromise patient safety for profit. Given that the community pharmacy in Nigeria functions like a primary healthcare setting, being the first point of access to healthcare by many Nigerians (10), the government must prioritize investing in these settings. Although currently, the government dedicates just about 5% of the national budget to healthcare, which is grossly inadequate to fund the public healthcare system, increasing this allocation to meet the Abuja declaration of 15% (10) could transform healthcare delivery nationwide and may also provide an opportunity to support privately owned community pharmacies. To improve renumeration and working conditions for community pharmacists, the ACPN in collaboration with the PCN needs to establish a standardized minimum (hourly) wage specifically for community pharmacists based on highest qualifications, experience, and responsibilities, and advocate for adherence to this minimum wage across the sector. Additionally, we recommend that ACPN publish regular surveys to increase salary transparency for community pharmacists to empower community pharmacists in negotiating for fair compensation. International pharmacy chains could establish joint partnerships with local pharmacies in Nigeria to facilitate capital investments and transfer of operational expertise. Additionally, the ACPN could implement anonymous online reporting systems where pharmacists facing coercion or threats to engage in unethical practices can safely report such incidents. These confidential online platforms are cost-effective and could encourage disclosure of managerial misconduct that compromises professional standards, while the risk of being reported may serve as a deterrent to managers pressuring staff into unethical practices. Following reports, ACPN could investigate cases and advocate for improved workplace conditions and ethical management practices.

Regarding pharmacy practice regulation, Nigeria faces significant challenges, primarily stemming from limited national capacity to implement core regulatory functions. The study by Usar and Bukar (15) reveals widespread issues with pharmacy regulation by PCN including inadequate funding, insufficient staffing, poor logistics, and infrastructural deficits that hamper effective monitoring of community pharmacy outlets. PCN operates with fixed budgetary allocations from the government that do not meet operational needs, resulting in irregular inspections and weak enforcement. Reinforcing compliance with established practice standards through fines and charges may increase funding for regulatory activities while providing compelling motivation for non-compliant pharmacists to improve their practice. Furthermore, educating the public to demand ethical pharmacy services while reporting violators may enhance customer-driven regulation of pharmacy practices. Adequate legislative support in resolving legal battles affecting pharmaceutical regulation is also essential to promote pharmacy regulation (15). By ensuring consequences for unethical practices through fines and charges, pharmacy managers may be discouraged from pressuring community pharmacists to engage in unethical practices. Additionally, enhanced funding for regulatory activities may enable PCN to effectively identify and take action against pharmacies that are not properly owned, managed, or operated by qualified pharmacists as well as clamp down on PPMVs who dispense antimicrobials unethically.

While regulatory enforcement through penalties and oversight must form the foundation of ethical practice standards, complementary educational approaches are equally essential to create sustainable change in pharmacy practice. Improving awareness and training on professional ethics and the importance of ethical practice, as well as advocating for increased reference to the PCN code of ethics are strategies that could enhance professionalism. To improve training on ethics, the PCN could establish formal partnerships with schools of pharmacies to enhance ethical education in pharmacy curricula. These collaborations could develop comprehensive ethics modules and workshops that utilize simulation exercises and case-studies to address real-world ethical dilammas pharmacists commonly encountered in Nigeria’s challenging practice environment. Additionally, PCN should establish ethics-focused continuous professional development modules for practicing pharmacists that address ethical challenges and reinforce professional standards. Certification in these modules could be mandated as a prerequisite for license renewal. This dual approach would ensure both new graduates and practicing professionals maintain strong ethical knowledge and practice. To reduce implementation costs, PCN could leverage volunteer pharmacists in developing these course modules and publish the modules online. These resource-efficient strategies would address funding limitations while maximizing ethical practice reinforcement across Nigeria’s community pharmacy landscape, including in remote and underserved regions.

Lastly with just about half of pharmacists reported disposing of expired or damaged medication through NAFDAC, there is need for more collaboration between NAFDAC, PCN and ACPN for a more efficient means of medicines disposal. PCN and NAFDAC should provide pharmacists with clear information on the importance of proper drug disposal channels to mitigate environmental and health risks. Utilizing online communication channels, PCN meetings, and side sessions at conferences to educate community pharmacists on proper drug disposal are cost-effective ways to raise awareness and promote better practices. The cost of disposing expired medicines through NAFDAC, including planning, logistics, and transportation costs, will be borne by the community pharmacists and may pose a major barrier to such activities (36). The ACPN could partner with NAFDAC to coordinate the collection of expired and damaged medicines from community pharmacists across a region, securing a discount for bulk disposal, thus helping to reduce costs.

## Limitations

Although the parallel mixed-method study design bolstered this research by integrating qualitative and quantitative data, this study is still subject to certain limitations. The data collection method used was convenience sampling, meaning that the sample may not be a true representation of the entire population, limiting the generalisability of our findings. Also, the data collection tool was disseminated online to reduce costs and efficiently utilise manpower, however, this approach may have excluded pharmacists without internet access during the data collection period. We acknowledge this limitation and encourage future research assessing pharmacy practice to explore both online and offline data collection methods.

Responses from this study did not come from all 36 states in Nigeria, hence, our findings may not be generalisable to the National population. It is likely that the mass exodus of over 7,000 pharmacists from Nigeria (37), the uneven distribution of community pharmacists across the nation, and the withdrawal of pharmacists from Boko-haram and other conflict-affected states may have contributed to limited responses from some states. The use of self-administered questionnaire may have limited community pharmacists’ ability to express in-depth experiences. Interviews may provide a better opportunity for self-expression regarding the drivers of unethical practices. Future research could explore qualitative approaches to assess pharmacy practices, such as simulated client method (SCM) and key informant interviews or discussion sessions, to assess the influence of reported factors and identify other unreported drivers of unethical practices. Employing a questionnaire as the primary data collection method may have introduced the risk of the Hawthorne effect, where pharmacists might have altered their responses to align with socially acceptable practices rather than reporting their actual behaviors. However, we mitigated this by ensuring anonymity and confidentiality, which helped respondents feel more comfortable providing honest responses. Also, the comparison of both quantitative and qualitative responses revealing consistent patterns reported across several states with specific examples of ethical challenges bolstered the credibility of our findings.

## Conclusion

The findings of this study highlight that while most community pharmacists adhere to ethical guidelines in providing pharmaceutical care, there are still lapses impeding optimal care delivery. Limited interprofessional collaborations among healthcare providers, poor regulations, and deplorable working conditions that prioritize profit over professionalism are salient drivers influencing unethical practices among community pharmacists.

We recommend that strengthening regulator frameworks while providing sanctions for defaulters, enhancing work conditions, offering incentives for professionalism, and reducing profit-driven pressures on pharmacists could significantly improve ethical standards in community pharmacy practice in Nigeria.

## Data Availability

The raw data supporting the conclusions of this article will be made available by the authors, without undue reservation.
